# Dynamic arm study: quantitative description of upper extremity function and activity of boys and men with duchenne muscular dystrophy

**DOI:** 10.1186/s12984-017-0259-5

**Published:** 2017-05-26

**Authors:** Mariska M. H. P. Janssen, Jaap Harlaar, Bart Koopman, Imelda J. M. de Groot

**Affiliations:** 1 Department of Rehabilitation, Radboud University Medical Center, Donders Centre for Neuroscience, Reinier Postlaan 4, 6525 GC Nijmegen, The Netherlands; 2 Department of Rehabilitation Medicine, VU University Medical Center, MOVE Research Institute, Amsterdam, The Netherlands; 3 Department Biomechanical Engineering, University of Twente, Enschede, The Netherlands

**Keywords:** Duchenne muscular dystrophy, Upper limb, 3D motion analysis, Surface electromyography, Muscle ultrasound, Muscle torque

## Abstract

**Background:**

Therapeutic management of upper extremity (UE) function of boys and men with Duchenne Muscular Dystrophy (DMD) requires sensitive and objective assessment. Therefore, we aimed to measure physiologic UE function of healthy subjects and DMD patients in different disease stages, and to evaluate the relation between these physiologic measures and functional UE scales.

**Methods:**

Twenty-three DMD patients and twenty healthy controls (7–23 years) participated in this explorative case–control study. Maximal muscle torque, maximal and normalized surface electromyography (sEMG) amplitudes, muscle thickness, echogenicity and maximal passive and active joint angles were measured. At activity level, Brooke upper extremity rating scale and the Performance of Upper Limb (PUL) scale were used.

**Results:**

Outcome measures related to proximal UE function could discriminate between disease stages. Increased normalized sEMG amplitudes were found in patients, even in early disease stages. Maximal active joint angles showed the strongest relation to Brooke scale (*R* ^*2*^ = 0.88) and PUL scale (*R* ^*2*^ = 0.85).

**Conclusions:**

The decline of muscle functions precedes the decline in performance of UE activities, and therefore may play a role in early detection of UE limitations. Increased sEMG levels demonstrate that DMD patients use more of their muscle capacity compared to healthy subjects, to perform daily activities. This might result in increased fatigability. Active maximal joint angles are highly related to functional scales, so preserving the ability to use the full range of motion is important for the performance of daily activities. Close monitoring of active joint angles could therefore help in starting interventions that minimize functional UE decline in DMD patients timely.

## Background

Duchenne Muscular Dystrophy (DMD) is a x-linked neuromuscular disorder with an incidence of 1:5,000 male newborns [1]. The disorder is characterized by a progressive loss of muscle strength, starting in the pelvic girdle, however, in later stages all muscles become affected. Boys with DMD become non-ambulant around the age of 10 years when untreated, and around the age of 13 years when treated with corticosteroids [2]. Arm function is already affected at this age [3, 4]. Although there is no curative treatment for DMD, life expectancy is rapidly increasing due to medical interventions [5, 6]. This means that boys and men with DMD have to live longer with their functional limitations and thus maintaining upper extremity (UE) function and measuring changes in UE function are increasingly important.

Loss of UE function can be delayed by several years by using corticosteroid treatment [7–10]. Physical exercise programs have also been found to be beneficial for retaining UE function [11–13]. However, in the long term, interventions that compensate for loss of UE function are still needed, for example arm supports, which reduce the effort that is needed to perform activities. To develop and evaluate such interventions, more insight in the upper extremity is needed. Insights on both International Classification of Functioning, Disability and Health (ICF) [14] function and structure level, and ICF activity level are necessary in order to unravel the mechanisms of UE decline.

The primary aim of this study is to give a quantitative description of UE functioning during a variety of meaningful UE task in boys and men with DMD in different stages of the disease, in comparison to their healthy peers. The secondary aim is to evaluate the relation between physiologic and structural UE functions and functional UE scales.

## Methods

### Population

The study population consisted of 23 boys and men with DMD and 20 healthy boys and men. DMD patients were included if they were older than 6 years, had a DNA established DMD diagnosis, and had a Brooke scale [15] of 1–5, meaning that they were able to use their hands functionally. Patients were recruited through the Radboud University Medical Center (Radboudumc) outpatient clinic and by an advertisement on the website of the Dutch DMD patient organization (“Duchenne Parent Project”). Healthy subjects over 6 years, without UE mobility limitations, were included from schools in the neighborhood of the Radboudumc in the city of Nijmegen. This study was approved by the medical ethical committee Arnhem–Nijmegen, the Netherlands (Registration number 2012/135, NL nr.: 39126.091.12). Informed consent was obtained from all participants and from their parents when the subjects were under 18 years of age.

### Outcome measures

#### Participant characteristics

The following participant characteristics were collected based on self-reports: age, arm preference, weight, height, year of diagnosis, wheelchair confinement and, if applicable the age of wheelchair confinement, and the occurrence of scoliosis.

#### Functional UE scales

Functional UE scales used in this study were: “Brooke upper extremity rating scale [15]” and the “Performance of Upper Limb (PUL) scale [16]”. These functional scales measured participants’ activity level. PUL items were performed once. Based on the score of the entry item, some subjects only performed a specific subset of the PUL. Sum scores of the 3 dimensions (high level shoulder, mid level elbow, distal wrist and hand) and the total sum score were calculated.

#### Muscle torques and surface electromyography

Muscle torques and surface electromyography (sEMG) signals were recorded of 7 different upper extremity muscles (Trapezius (descending part), Biceps Brachii (long head), Triceps Brachii (long head), Deltoid (lateral part), Pectoralis Major (clavicular head), wrist flexors and wrist extensors). Muscle torques were measured using a static frame myometer, consisting of a KAP-E Force Transducer, measurement range 0.2–2000 N (Angewandte System Technik, Dresden, Germany), and a height and position adjustable frame (designed and custom made by mechanical engineers from the VU medical centre, Amsterdam, the Netherlands). Wireless sEMG signals (Zerowire EMG, Aurion, Italy) were recorded with a sample frequency of 1000 Hz. Disk-shaped Ag–AgCL ARBO ECG electrodes (Tyco Healthcare, Neustadt, Germany) were placed at an inter electrode distance of 24 mm. Testing and electrode positions were based on literature [17, 18]. To make the measurement protocol more suitable for DMD patients, as they were often in a wheelchair or had joint contractures, we slightly adapted some of the testing positions. sEMG data were filtered using a 4^th^ order band pass filter between 20 and 450 Hz, where after the signal was rectified and low pass filtered (3 Hz) to obtain the linear envelope [19, 20]. Torque data were filtered using a 3 Hz low pass filter of the 4^th^ order.

All subjects performed two maximal voluntary isometric contractions (MVICs) to determine the maximal muscle torque and corresponding sEMG amplitude. If the examiner was not confident that a maximal effort was made, the measurement was repeated. The maximal value out of the two correct attempts was used for further data analysis. Normalized sEMG amplitudes were calculated for the performance of single joint movements and PUL items. Normalized sEMG amplitude was defined as the maximum sEMG amplitude that was reached during a movement as a percentage of the maximal amplitude of the same muscle during MVIC.

Data was processed with custom-written Matlab (Matlab® version R2014b, Mathworks, Natick, USA) routines.

#### Quantitative muscle ultrasound

Ultrasounds images of 6 upper extremity muscles (Trapezius, Biceps Brachii, Triceps, Deltoid, wrist flexors and wrist extensors) were recorded using a Z.One PRO Ultrasound System (Zonare Medical Systems, Mountain View, California, USA), with a L10-5 transducer. Three ultrasound recordings were made, at a depth of 4 cm, to calculate echogenicity (greyscale) and one recording, with no predefined depth, was made to determine the muscle thickness. Echogenicity is the extent to which a structure reflects ultrasound of a surface with high echogenicity indicating that more ultrasound is reflected, for example when high levels of fatty and connective tissue are present in a muscle. Ultrasound images were analyzed with computer-assisted greyscale histogram analysis, using custom software developed at Radboudumc (QUMIA). Echogenicity was determined by calculating the grayscale in the upper 1/3^rd^ of the region of interest (the region that included as much muscle mass as possible without bone and fascia) in each muscle [21]. The average echogenicity out of 3 measurements was used for further analysis. Muscle thickness was determined by calculating the distance between two electronic calipers at standardized positions. Thickness of the Trapezius was measured between the deep and superficial fascia of the upper part of the Trapezius muscle. Thickness of the Deltoid, Biceps (combined with Brachialis) and Triceps muscles were measured between the humerus and the superficial fascia. Forearm flexor (Flexor Carpi Radialis) thickness was measured between a horizontal reference line at the height of the radius and the superficial fascia. Forearm extensors thickness was measured between the middle end of the radius and the superficial fascia.

Ultrasound results were compared to muscle specific reference values and expressed as Z-scores (representing the number of standard deviations from the mean) [22]. Reference values for calculation of the Z-scores were obtained from 60 healthy subjects using the same measurement protocol and ultrasound device (manuscript in preparation). Echogenicity and muscle thickness were corrected for age, weight and height if necessary using the method described by Scholten et al. [23].

#### Three dimensional motion analysis

**Fig. 1:**
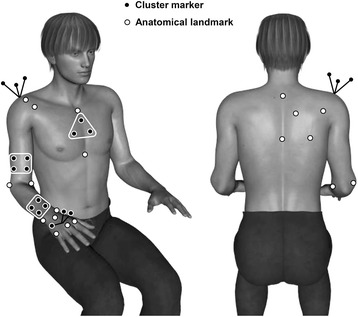
Marker positions. Positions of cluster markers (*black center*) and anatomical landmarks (*white center*)

**Fig. 2:**
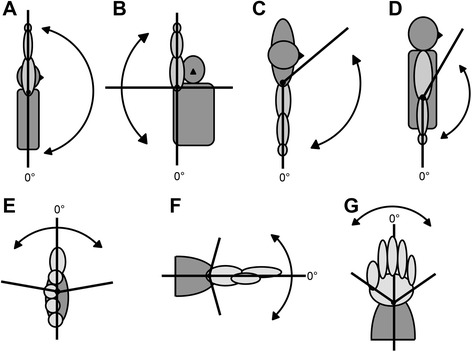
Single joint movements. **a** shoulder flexion, (**b**) shoulder abduction, (**c**) shoulder adduction (in the horizontal plane), (**d**) elbow flexion and extension, (**e**) forearm pronation and supination, (**f**) wrist flexion and extension and (**g**) wrist ulnar and radial deviation

Three dimensional motion analysis, using the kinematic model of Jaspers et al. [24] (Fig. 1), was performed with an 8 camera VICON motion analysis system (Oxford Metrics, Oxford, UK). After marker placement and anatomical landmark identification, maximal passive joint angles were determined for: ‘shoulder abduction’ , ‘elbow flexion and extension’ , ‘pro- and supination of the lower arm’, ‘wrist flexion and extension’ and ‘wrist ulnar and radial deviation’. Maximal active joint angles were determined for the same movements and also for ‘shoulder flexion’ and ‘shoulder adduction (in the horizontal plane)’ (Fig. 2). Some subjects did not perform all single joint movements as they were unable to perform the movements. All passive and active movements were performed 3 times at a controlled movement velocity.

Joint-kinematics were calculated using BodyMech (http://www.bodymech.nl) and additional custom-written Matlab routines. Kinematic data were filtered using a 4^th^ order low pass filter of 20 Hz. Per movement, the minimal and maximal joint angles were determined. The average maximal joint angle over three measurements was used for further data analysis.

### Statistical analysis

Median values and ranges were used to describe the continuous participant characteristics and percentages were used to describe categorical participant characteristics. Wilcoxon rank sum tests were used to compare outcome measure sum scores between healthy subjects and DMD patients. Kruskal-Wallis tests were used to test for differences between DMD patients in different Brooke scales. To gain insight in the relation between functional UE scales (Brooke and PUL scale) and physiologic UE function (muscle torque, sEMG, echogenicity, muscle thickness, passive and active joint angles) we calculated the coefficient of determination (R^2^) between the sum scores, or average scores for echogenicity and muscle thickness, of these outcome measures. The sum scores were calculated by adding the results of all values within one outcome measure. If one or more values were missing, the sum score was also reported as missing. If values were missing because patients were physically unable to perform the activity a score of 0 was used for the calculation of the sum scores. SPSS Statistics Version 20 (IBM, Somers, USA) was used for statistical analysis.

## Results

The median age of healthy subjects was 14.0 (range 7.4–23.4) years and the median age of DMD patients was 14.9 (range 8.1–21.7) years (Table 1). About 90% of the participants was right handed. The median age at diagnosis was 3.75 years (range 0–7 years) and 74% of the patients was non-ambulant. Thirteen percent of the patients had a mild scoliosis, and 22% had a severe scoliosis, of which 40% was surgically corrected. Corticosteroids were used by 74% of the patients, while 13% stopped using and 13% never used corticosteroids. Of the corticosteroid users, 12% used Deflazacort on a daily basis and 88% uses Prednisone/Prednisolone on a 10-days-on/10-days-off basis. Dosages vary between 4 and 45 mg.

**Table 1:** Participant characteristics

Variable	Healthy	DMD Brooke 1	DMD Brooke 2	DMD Brooke 3	DMD Brooke 4	DMD Brooke 5
N	20	5	8	4	3	3
Age (median, range)	14.0 (7.4–23.4)	11.1 (8.0–16.0)	12.4 (8.7–15.8)	15.8 (12.6–16.9)	17.1 (17.0–18.4)	18.2 (17.8–21.7)
BMI	19.1 (15.7–24.4)	21.9 (17.7–26.4)	20.7 (18.7–30.8)	23.0 (16.5–26.0)	17.6 (10.0–21.4)	22.5 (19.4–24.6)
Hand preference						
Right handed (%)	90	100	87.5	75	100	100
Left handed (%)	10	0	12.5	25	0	0
Age of diagnosis (median, range)	-	2.0 (0.0–5.0)	4.0 (2.5–7.0)	4.0 (2.5–6.0)	1.5 (0.0–6.0)	1.0 (0.0–6.0)
Percentage wheelchair confined (%)	-	0	87.5	100	100	100
Age wheelchair confined (median, range)	-	-	10 (7–13)	10 (8–10)	11 (10–11)	9 (8–10)
Scoliosis						
No (%)	-	75	87.5	75	33	0
Mild (%)	-	25	12.5	0	0	33
Severe (%)	-			25	67	67
Scoliosis correction (%)	-	0	0	0	33	67
Corticosteroid use						
No (%)	-	0	12.5	25	33	0
Not anymore (%)	-	0	0	25	0	67
Yes (%)	-	100	87.5	50	67	33

Statistically significant differences between healthy subjects and DMD patients were seen in all outcome measures except muscle thickness, as all the Z-values for muscle thickness were between −2 and 2 (Table 2). In addition, differences between patients in different Brooke scales were present in most proximal muscles and movements requiring proximal muscles. PUL scores in all domains differed between DMD patients in different Brooke scales.

**Table 2:** Average UE outcome measures scores for healthy subjects and DMD patients in different Brooke scales

	Healthy			DMD Brooke 1			DMD Brooke 2			DMD Brooke 3			DMD Brooke 4			DMD Brooke 5				
	N	Mean	(95% CI)	N	Mean	(95% CI)	N	Mean	(95% CI)	N	Mean	(95% CI)	N	Mean	(95% CI)	N	Mean	(95% CI)	*P*-value healthy/patient	*P*-value Brooke scale
Functional UE scales																				
Performance of Upper Limb Scale																				
PUL shoulder	20	16	(.;.)	5	13	(10;15)	8	6	(3;10)	4	0	(.;.)	3	0	(.;.)	2	0	(.;.)	**0,000**	**0,002**
PUL elbow	20	32	(.;.)	5	32	(31;32)	8	27	(22;32)	4	15	(6;24)	3	10	(4;15)	2	5	(−15;24)	**0,000**	**0,002**
PUL wrist	20	24	(.;.)	5	24	(23;24)	8	22	(21;23)	4	22	(21;23)	3	20	(15;26)	2	20	(.;.)	**0,000**	**0,014**
Sum score^a^	20	78	(.;.)	5	74	(71;76)	8	60	(52;68)	4	41	(31;50)	3	32	(21;43)	2	26	(6;45)	**0,000**	**0,001**
Physiologic UE outcome measures																				
Maximal muscle force (N)																				
Trapezius	20	476	(344;609)	5	147	(62;233)	8	109	(85;134)	3	138	(−4;280)	3	59	(29;90)	3	40	(−6;85)	**0,000**	**0,012**
Biceps	20	178	(139;217)	5	37	(27;47)	8	27	(19;34)	3	21	(17;24)	3	7	(−3;17)	3	5	(−2;12)	**0,000**	**0,003**
Triceps	20	152	(121;182)	5	27	(21;33)	8	17	(11;23)	3	13	(4;21)	3	8	(2;14)	2	7	(−40;53)	**0,000**	**0,011**
Deltoid	20	83	(64;102)	5	32	(24;40)	8	21	(14;27)	3	21	(17;26)	2	15	(3;26)	2	7	(−21;35)	**0,000**	**0,020**
Pectoralis major	20	199	(156;242)	5	69	(49;89)	8	45	(30;60)	3	40	(21;58)	3	14	(−6;34)	3	15	(7;23)	**0,000**	**0,003**
Wrist flexors	20	127	(102;152)	5	29	(14;44)	7	27	(11;42)	3	26	(−38;90)	3	19	(−9;47)	2	14	(−32;61)	**0,000**	0,386
Wrist extensors	20	120	(94;146)	5	32	(13;51)	7	30	(14;45)	3	30	(−20;80)	3	8	(−8;24)	2	14	(−19;47)	**0,000**	0,061
Sum score	20	1334	(1038;1631)	5	373	(271;474)	7	278	(212;344)	3	288	(16;561)	2	147	(−81;374)	2	110	(−192;412)	**0,000**	**0,024**
Maximal muscle torque (Nm)																				
Trapezius	20	86,4	(57,3;115,5)	5	24,6	(11,5;37,7)	8	16,2	(11,9;20,5)	3	24,8	(−6,0;55,5)	3	8,5	(2,4;14,6)	3	7,2	(−2,6;16,9)	**0,000**	**0.016**
Biceps	20	45,8	(33,8;57,7)	5	7,2	(4,8;9,6)	8	5,9	(4,1;7,6)	3	5,0	(2,8;7,2)	3	1,7	(−1,2;4,5)	3	1,3	(−0,7;3,3)	**0,000**	**0.010**
Triceps	20	38,2	(28,9;47,5)	5	5,4	(4,0;6,8)	8	3,8	(2,3;5,4)	3	3,2	(1,2;5,2)	3	1,8	(−0,4;4,0)	2	1,6	(−11,1;14,3)	**0,000**	**0.024**
Deltoid	20	41,4	(29,8;52,9)	5	11,1	(8,4;13,7)	8	8,6	(5,5;11,6)	3	9,5	(7,9;11,2)	2	5,6	(4,9;6,2)	2	2,7	(−10,0;15,4)	**0,000**	0.071
Pectoralis major	20	55,2	(41,2;69,3)	5	13,6	(9,4;17,7)	8	10,2	(6,9;13,6)	3	10,3	(7,0;13,5)	3	3,4	(−3,0;9,8)	3	3,8	(1,4;6,2)	**0,000**	**0.010**
Wrist flexors	20	8,4	(6,4;10,4)	5	2,9	(−0,5;6,2)	7	2,0	(0,5;3,4)	3	1,5	(−1,9;4,9)	3	1,1	(−0,9;3,1)	2	0,8	(−1,2;2,7)	**0,000**	0.311
Wrist extensors	20	7,9	(5,8;10,0)	5	3,2	(−0,8;7,2)	7	2,1	(0,7;3,6)	3	1,8	(−0,7;4,3)	3	0,4	(−0,3;1,1)	2	0,8	(−1,2;2,7)	**0,000**	**0.027**
Sum score	20	283,2	(207,5;358,9)	5	68,0	(48,1;87,8)	7	48,1	(34,0;62,2)	3	56,1	(19,0;93,2)	2	25,5	(−35,5;86,5)	2	19,5	(−65,0;103,9)	**0,000**	**0.026**
Maximal sEMG amplitude (mV)																				
Trapezius	20	0,38	(0,27;0,50)	5	0,19	(0,03;0,35)	8	0,16	(0,09;0,24)	4	0,09	(0,05;0,13)	3	0,07	(−0,04;0,19)	3	0,02	(0,00;0,03)	**0,000**	**0,032**
Biceps	20	0,89	(0,73;1,05)	5	0,19	(0,01;0,37)	8	0,15	(0,09;0,21)	4	0,11	(−0,06;0,28)	3	0,07	(−0,01;0,15)	2	0,02	(−0,18;0,21)	**0,000**	0,096
Triceps	20	0,61	(0,48;0,74)	5	0,10	(0,03;0,16)	8	0,07	(0,05;0,09)	4	0,03	(0,02;0,03)	3	0,04	(−0,02;0,09)	3	0,02	(−0,02;0,05)	**0,000**	**0,004**
Deltoid	20	0,61	(0,50;0,72)	5	0,17	(0,12;0,22)	8	0,20	(0,10;0,30)	3	0,07	(.;.)	2	0,06	(−0,14;0,25)	3	0,06	(−0,06;0,19)	**0,000**	**0,023**
Pectoralis major	20	0,57	(0,42;0,73)	5	0,12	(0,04;0,20)	8	0,08	(0,04;0,11)	4	0,03	(0,00;0,05)	3	0,05	(−0,04;0,14)	3	0,01	(0,00;0,03)	**0,000**	**0,007**
Wrist flexors	20	0,26	(0,19;0,32)	5	0,06	(0,04;0,09)	8	0,07	(0,04;0,11)	4	0,05	(0,02;0,07)	3	0,03	(0,01;0,05)	3	0,04	(0,02;0,06)	**0,000**	0,056
Wrist extensors	20	0,47	(0,38;0,56)	5	0,13	(0,06;0,20)	8	0,18	(0,08;0,28)	4	0,10	(0,05;0,15)	3	0,12	(−0,10;0,34)	3	0,05	(−0,03;0,12)	**0,000**	0,090
Sum score	20	3,79	(3,28;4,30)	5	0,96	(0,57;1,34)	8	0,92	(0,71;1,13)	3	0,39	(0,32;0,46)	2	0,51	(−1,08;2,09)	2	0,21	(−0,62;1,03)	**0,000**	**0,011**
Z-scores Echogenicity (1/3 ROI)																				
Trapezius	16	0,37	(−0,27;1,02)	5	3,24	(0,89;5,59)	8	3,23	(1,35;5,11)	4	2,90	(−0,60;6,40)	3	6,47	(5,02;7,91)	2	3,19	(2,61;3,76)		0,093
Deltoid	16	0,36	(−0,21;0,94)	5	5,07	(4,17;5,96)	8	5,48	(4,03;6,92)	4	4,53	(0,39;8,67)	3	9,30	(8,39;10,21)	3	7,64	(5,18;10,10)		**0,016**
Biceps	16	0,14	(−0,30;0,59)	5	5,73	(3,39;8,06)	8	5,74	(4,44;7,04)	4	6,35	(3,91;8,78)	3	7,38	(5,88;8,88)	3	6,96	(3,08;10,84)		0,418
Triceps	16	0,23	(−0,43;0,88)	5	4,92	(3,47;6,37)	8	7,20	(5,58;8,82)	4	7,51	(5,19;9,83)	3	6,73	(4,44;9,03)	3	7,39	(4,61;10,17)		0,114
Wrist flexors	20	0,47	(−0,04;0,97)	5	3,21	(2,57;3,84)	8	3,63	(2,48;4,78)	4	4,32	(3,20;5,44)	3	5,19	(2,77;7,60)	2	5,02	(0,12;9,91)		0,065
Wrist extensors	14	0,24	(−0,07;0,55)	5	2,91	(1,31;4,52)	8	3,03	(1,56;4,50)	4	2,97	(1,59;4,36)	3	4,57	(4,12;5,03)	2	4,68	(3,79;5,57)		0,372
Mean score	14	0,34	(0,01;0,68)	5	4,18	(3,08;5,28)	8	4,72	(3,66;5,77)	4	4,77	(2,83;6,70)	3	6,61	(5,79;7,43)	2	5,79	(2,61;8,97)		0,053
	Healthy			DMD Brooke 1			DMD Brooke 2			DMD Brooke 3			DMD Brooke 4			DMD Brooke 5				
	N	Mean	(95% CI)	N	Mean	(95% CI)	N	Mean	(95% CI)	N	Mean	(95% CI)	N	Mean	(95% CI)	N	Mean	(95% CI)	*P*-value healthy/ patient	*P*-value Brooke scale
Z-scores Muscle Thickness																				
Trapezius	15	−0,04	(−0,83;0,74)	4	0,54	(−1,15;2,23)	7	1,08	(−0,50;2,65)	2	1,53	(−17,15;20,21)	2	−1,40	(−14,49;11,69)	2	0,29	(−8,67;9,24)		0,401
Deltoid	16	0,02	(−0,77;0,80)	4	0,82	(−1,05;2,70)	4	0,88	(−0,96;2,71)	1	0,02	(.;.)	3	−1,86	(−5,64;1,91)	3	1,47	(−5,05;7,99)		0,289
Biceps	16	0,73	(−0,67;2,14)	5	−0,80	(−2,75;1,14)	7	−1,64	(−3,06;−0,22)	2	−1,61	(−35,98;32,77)	1	−0,26	(.;.)	2	1,14	(−26,69;28,97)		0,684
Triceps	16	−0,22	(−0,85;0,41)	5	0,28	(−1,40;1,96)	5	−1,01	(−2,27;0,25)	0	.	(.;.)	2	−1,72	(−5,08;1,65)	0	.	(.;.)		0,087
Wrist flexors	16	−0,31	(−0,96;0,33)	5	−1,08	(−1,94;−0,23)	7	−1,10	(−1,87;−0,33)	2	−1,86	(−11,70;7,99)	2	−1,46	(−9,97;7,05)	1	0,83	(.;.)		0,466
Wrist extensors	14	0,39	(−0,31;1,08)	5	−0,04	(−0,70;0,61)	8	0,88	(−0,34;2,10)	4	0,76	(−0,49;2,00)	3	−0,64	(−5,03;3,75)	1	0,97	(.;.)		0,494
Mean score	13	0,13	(−0,69;0,96)	3	−0,17	(−1,13;0,79)	4	−0,48	(−1,41;0,45)	0	.	(.;.)	1	−0,41	(.;.)	0	.	(.;.)		0,757
Passive maximal joint angles (°)																				
Shoulder abduction	20	149	(142;155)	5	155	(145;164)	8	151	(136;166)	4	151	(134;168)	3	128	(97;160)	2	114	(63;165)	0,420	**0,046**
Elbow flexion	20	145	(143;148)	5	133	(126;139)	8	138	(131;145)	4	134	(116;151)	3	135	(114;155)	3	129	(123;134)	**0,000**	0,595
Elbow extension	20	4	(−1;9)	5	4	(−7;15)	8	23	(15;32)	4	35	(−11;80)	3	50	(−45;146)	3	58	(18;98)	**0,001**	**0,031**
Pronation	20	94	(85;103)	5	70	(37;103)	8	56	(33;78)	4	73	(16;129)	3	76	(32;119)	3	57	(19;94)	**0,001**	0,665
Supination	20	−59	(−68;−50)	5	−56	(−79;−34)	8	−50	(−73;−27)	4	−7	(−64;50)	3	−35	(−152;83)	3	−9	(−75;56)	0,072	0,097
Wrist flexion	20	−76	(−82;−71)	5	−65	(−88;−42)	8	−63	(−83;−44)	4	−60	(−78;−42)	3	−32	(−127;63)	3	−43	(−73;−12)	**0,001**	0,187
Wrist extension	20	81	(72;91)	5	65	(54;76)	8	84	(74;95)	4	55	(−6;116)	3	65	(25;106)	3	29	(−41;99)	**0,019**	**0,047**
Radial deviation	20	−34	(−38;−30)	5	−37	(−54;−21)	8	−41	(−53;−30)	4	−29	(−52;−5)	3	−19	(−97;59)	3	−16	(−34;3)	0,893	0,188
Ulnar deviation	20	31	(28;34)	5	25	(9;42)	8	21	(0;43)	4	19	(13;25)	3	30	(−29;89)	3	20	(−13;52)	**0,047**	0,854
Sum score	20	666	(643;690)	5	603	(525;680)	8	581	(519;644)	4	492	(265;720)	3	470	(248;691)	2	398	(−381;1177)	**0,000**	0,109
Active maximal joint angles (°)																				
Shoulder flexion	20	140	(134;145)	5	142	(129;154)	8	126	(104;148)	4	36	(−3;75)	0	.	(.;.)	0	.	(.;.)	**0,003**	**0,001**
Shoulder abduction	20	141	(134;148)	5	142	(121;163)	8	125	(101;149)	4	36	(−2;73)	0	.	(.;.)	0	.	(.;.)	**0,002**	**0,001**
Shoulder adduction^b^	20	129	(124;135)	5	128	(120;137)	8	93	(70;116)	4	19	(−41;78)	0	.	(.;.)	0	.	(.;.)	**0,000**	**0,001**
Elbow flexion	20	138	(135;141)	5	122	(110;133)	8	130	(123;137)	4	117	(95;139)	3	110	(16;204)	0	.	(.;.)	**0,000**	**0,035**
Elbow extension	20	7	(2;12)	5	9	(1;17)	8	29	(17;41)	4	49	(12;86)	3	40	(−24;104)	0	.	(.;.)	**0,007**	**0,006**
Pronation	20	80	(73;87)	5	59	(34;84)	8	62	(51;73)	4	67	(0;134)	3	50	(2;99)	3	13	(−44;71)	**0,000**	0,148
Supination	20	−46	(−52;−41)	5	−39	(−54;−24)	8	−33	(−52;−14)	4	9	(−47;65)	3	−10	(−90;71)	3	−5	(−28;18)	**0,001**	0,066
Wrist flexion	20	−67	(−72;−61)	4	−57	(−76;−37)	8	−57	(−76;−38)	4	−57	(−63;−52)	3	−16	(−124;92)	3	−36	(−59;−12)	**0,003**	0,114
Wrist extension	20	80	(73;86)	4	78	(48;108)	8	77	(67;86)	4	46	(−28;119)	3	66	(35;96)	3	20	(−49;90)	**0,027**	0,063
Radial deviation	20	−30	(−35;−24)	4	−39	(−53;−25)	8	−36	(−48;−24)	4	−29	(−43;−14)	3	−25	(−97;47)	3	−14	(−23;−6)	0,365	0,177
Ulnar deviation	20	31	(29;33)	4	29	(8;50)	8	26	(19;34)	4	19	(9;30)	3	23	(−15;61)	3	14	(−6;33)	**0,007**	0,334
Sum score	20	875	(846;903)	4	838	(782;894)	8	736	(658;814)	4	367	(176;557)	3	260	(151;369)	3	102	(−60;265)	**0,000**	**0,001**

^a^PUL sum scores is calculated including the score of the entry item (maximal score = 78)

^b^Shoulder adduction in the horizontal plane (Fig. 2.C.)

*P*-values healthy/patient show the differences between healthy subjects and patients. *P*-values Brooke scale show the differences between DMD patients in different Brooke scales. *P*-values > 0.05 indicate a statistical significant difference and are displayed bold. *P*-values healthy/patient are not shown for echogenicity and muscle thickness z-scores, as the z-scores already indicate the difference with a healthy reference population

**Fig. 3:**
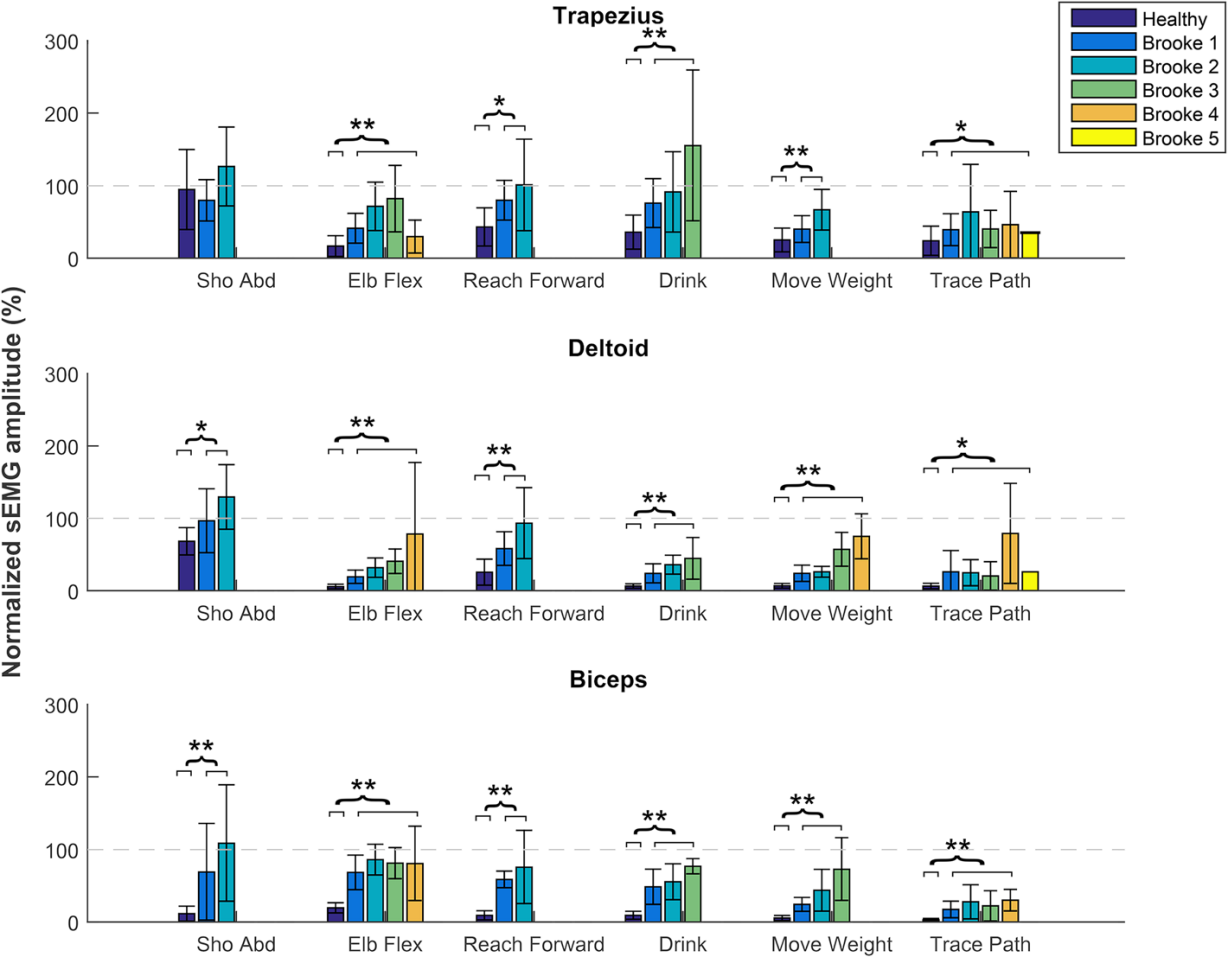
Normalized sEMG amplitudes. Normalized sEMG amplitudes of the Trapezius, Deltoid and Biceps Brachii muscles for 6 different upper extremity movements shown for healthy subject and DMD patients in different Brooke scales. Sho Abd = shoulder abduction; Elb Flex = elbow flexion; Reach Forward = reaching forward at shoulder level without weight (PUL item D); Drink = drinking from a full cup (200 g) (PUL item F); Move Weight = moving a 100 g weight (PUL item H); Trace Path = tracing a path (PUL item O)

**Fig. 4:**
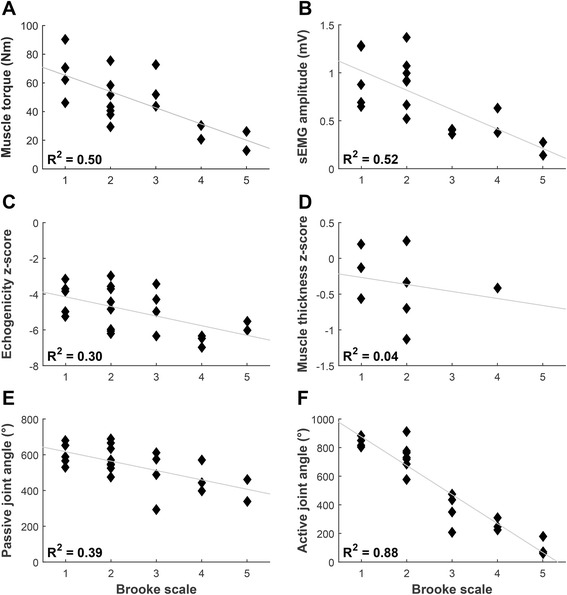
Correlations with Brooke scale. Correlations of DMD patients between Brooke score and (**a**) maximal muscle torque sum score; **b** maximal sEMG amplitude (MVIC) sum score; **c** mean inverse z-score of echogenicity (inverse z-scores were used so that lower scores indicate worse UE function); **d** mean z-score of muscle thickness; **e** maximal passive joint angle sum score; **f** maximal active joint angle sum score

**Fig. 5:**
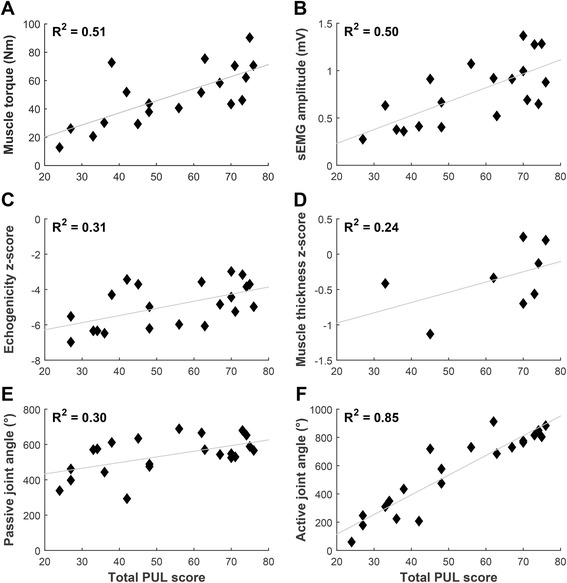
Correlations with PUL score. Correlations of DMD patients between total PUL score and (**a**) maximal muscle torque sum score; **b** maximal sEMG amplitude (MVIC) sum score; **c** mean inverse z-score of echogenicity (inverse z-scores were used so that lower scores indicate worse UE function); **d** mean z-score of muscle thickness; **e** maximal passive joint angle sum score; **f** maximal active joint angle sum score

Normalized sEMG amplitudes of DMD patients and healthy controls differed significantly for all movements and muscles, except for Trapezius activation during shoulder abduction (Fig. 3). Maximal active joint angle sum score shows the strongest correlations with Brooke scale (Fig. 4) and PUL score (Fig. 5) (*R* ^*2*^ of 0.88 and 0.85 respectively), followed by maximal muscle torque and maximal sEMG amplitude sum scores (*R* ^*2*^ > 0.5). Echogenicity and passive maximal joint angle sum scores explain about 30% of the variance of Brooke scale and PUL score.

**Fig. 6:**
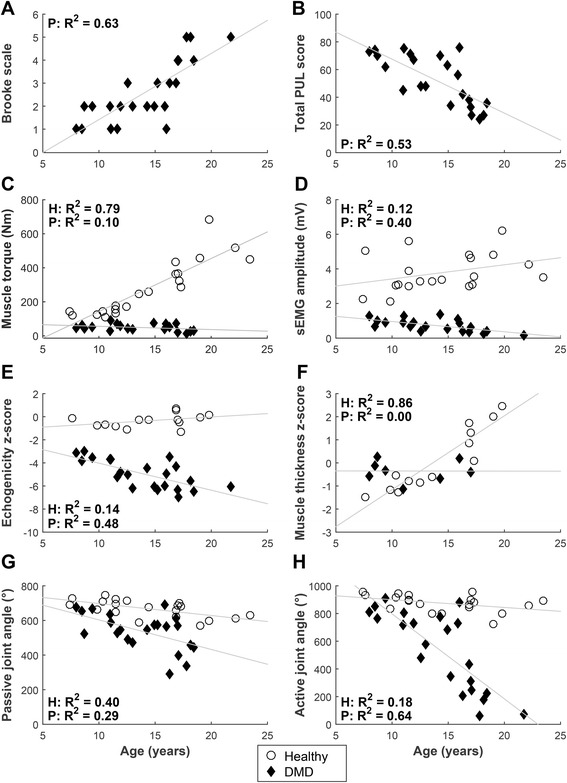
Correlations with age. Correlations of DMD patients and healthy subject between age and (**a**) Brooke scale; **b** total PUL score; **c** maximal muscle torque sum score; **d** maximal sEMG amplitude (MVIC) sum score; **e** mean inverse z-score of echogenicity (inverse z-scores were used so that lower scores indicate worse UE function); **f** mean z-score of muscle thickness; **g** maximal passive joint angle sum score; **h** maximal active joint angle sum score

In healthy subjects, a strong relation with age was present for maximal muscle torque sum score and mean muscle thickness z-score (Fig. 6, *R* ^*2*^ of 0.79 and 0.86 respectively). For DMD patients the strongest correlations with age were found for maximal active joint angle sum score and Brooke scale (*R* ^*2*^ of 0.64 and 0.63 respectively).

## Discussion

Our study provides new insights in the muscles and movements that are affected most in DMD patients, and how this relates to functional UE scales. This is vital information for clinical decision making, but can also be used in the development of new outcome measures in clinical trials.

Currently, functional scales such as the Performance of Upper Limb (PUL) scale and the Motor Function Measure (MFM) are used as the gold standard for quantifying UE limitations in DMD. These measures, however, are not able to cover the entire spectrum of DMD patients, as they have floor and ceiling effects [16]. Furthermore, they do not give insight in the underlying working mechanisms of the UE. Daily activities require sufficient strength of multiple muscle groups and motion in multiple joints. Therefore, functional scales give insight in problems that result from a combination of many different physiologic aspects of UE function.

Our study shows that muscle functions (i.e. maximal muscle torque, maximal sEMG amplitude and echogenicity z-scores) of DMD patients already deviate from healthy subjects in an early disease stage (i.e. Brooke 1). A similar reduction of muscle force/torque in young DMD patients has been reported in previously [3, 25–27]. Echogenicity z-scores of all muscles are above two thus differ significantly from the healthy reference population. This finding indicates that muscles are infiltrated with fatty and connective tissue, which is in line with the results of other studies [21, 28, 29]. Consequently, these outcome measures are of great importance for early detection of UE impairments, as activity scales cannot be used in the earliest disease stage due to ceiling effects. Early detection is important to start interventions early, for example physical exercise training, which is proven to be effective in delaying functional deterioration [11–13]. The current study shows that mainly proximal muscles and movements requiring proximal muscle activation are sensitive to detect differences of UE function and activity. Maximal muscle torques and maximal sEMG amplitudes of proximal muscles can also detect differences in the later disease stages (Brooke 4 and 5), even though the muscles cannot initiate movements anymore. This could be important for evaluating the effects of arm supports, or other interventions aimed at late stage DMD patients.

To identify which limitations are primarily responsible for the inability to perform activities, and how this relates to weakness in specific muscles, insight in single joint movements is important. Single joint movements consist of movements over one joint, which often can be related to the activation of one primary muscle. In clinical practice, the measurement of maximal active single joint angles can give more insight in the mechanism responsible for activity limitations. This statement is supported by the very strong relation we found between maximal active joint angles and PUL score (*R* ^*2*^ = 0.85).

Our results show that when the maximal Deltoid torque drops below approximately 10 Nm, DMD patients start to have difficulties lifting their arms. A maximal Biceps torque below approximately 5 Nm is related to restrictions in elbow motion. It is likely these are the minimum torques required to move the upper/lower arm against gravity, and could help to identify the suitable time to start using an arm support. Hence, regular assessment of deltoid and biceps torques may help clinicians plan interventions, anticipating functional decline.

We found that active and passive joint angles decline almost simultaneously. Therefore, we hypothesize that when a patient loses the ability to move a joint actively, the joint will be statically positioned for longer periods, which leads to contractures soon thereafter. This hypothesis is in line with the findings of McDonald et al. who showed the occurence of elbow flexion contractures appears to be related to static positions of the limb after wheelchair confinement [30]. Hence, we recommend to start interventions, such as stretching exercises, as soon as active joint angles start to decrease. In addition, we recommend stimulation of (supported) movement to limit static positioning and thereby prevent contracture formation [31].

A recent study has indicated that fatigue was strongly associated with health-related quality of life and that there should be a greater clinical focus on the reduction of fatigue [32]. In this study, we measured maximal sEMG amplitudes, which is a measure for the maximal muscle capacity. Normalized sEMG amplitudes show the percentage of this maximal muscle capacity needed to perform activities. When normalized sEMG amplitudes are high, a larger percentage of the muscle capacity is used, which leads to faster occurrence of fatigue [33]. Our results show that DMD patients use a larger percentage of their muscle capacity to perform movements and activities compared to healthy subjects, even in an early stage of the disease, and therefore might experience earlier and more fatigue. This increase in the percentage of muscle capacity is not only seen in prime movers, but also in secondary movers indicating the use of compensatory muscles to overcome loss of muscle strength. Future studies should try to determine normalized sEMG amplitudes and normalized sEMG median frequency during a fatigue protocol, in order to gain more insight in muscle fatigue of DMD patients compared to healthy controls.

Although most of our results are in line with existing literature, we also found some differences. The passive forearm supination angle of DMD patients in this study did not differ significantly (*p* = 0.072) from healthy subjects, as opposed to findings from Bartels et al. [27]. However, we found that the average forearm supination angles were reduced in patients from Brooke 3 onward, which is in line with the results of Bartels et al. who reported that 83% of the adult men with DMD had loss of supination [27]. As far as we are aware, the differences we found between healthy subjects and DMD patients for passive elbow flexion, forearm pronation, wrist flexion and ulnar deviation have not been reported before. Although the differences between healthy subjects and DMD patients are small, they could be of clinical relevance for the performance of daily UE activities [34–36].

Muscle ultrasounds are able to make distinction between different stages of DMD [21, 28] We, however, found that echogenicity is less strongly related to disease stage compared to maximal muscle torque and maximal sEMG amplitude. We expect that echogenicity, which is a measure for muscle degeneration, is less discriminative in the explored muscles because the ultrasound images are heavily affected by attenuation. This is especially true for the later disease stages, as an increased amount of fat and connective tissue in the muscles prevents the ultrasound from penetrating deeper layers of the muscle, which results in a darker picture and therefore lowers Z-scores. For the same reason muscle thickness could not be measured accurately in older patients.

### Study limitations

A limitation of this study is the relatively small number of patients in each group, especially in the latest disease stages. For this reason, post hoc comparisons between different disease stages were not performed. Furthermore, stratification of possible confounders, such as corticosteroid use and scoliosis, was not possible due to the small sample size. In addition, as this study is cross-sectional, we were unable to determine longitudinal changes of UE function. Therefore we recommend future UE studies to monitor changes of physiologic UE function over time in a cohort of patients. Nevertheless, our population is representative of the general DMD population, as the participant characteristics are comparable to literature.

A second limitation relates to our measurements of individual muscle strength. External muscle torque measurements, as we performed with the static frame myometer are unable to measure the maximal torque of isolated muscles. We attempted to mimic the activation of individual muscles as close as possible by choosing measurement positions that primarily required the activation of one muscle, the prime mover. We reported muscles torques as our primary outcome rather than muscle forces, which are more commonly used in literature. Muscle forces, however, do not account for the effect of lever arm, which we believe is more relevant in our study as we measured subjects in a wide age/height range [37]. For comparability we also reported maximal muscle forces in Table 2.

Finally, the use of normalized sEMG amplitudes has some limitations as well . The maximal sEMG amplitude (in MVIC) can be influenced by pain, fear of pain, restrictions in the range of motion and/or motivation [18]. As a result normalized sEMG amplitudes over 100% MVIC were sometimes seen. This underperformance during MVIC measurements could affect both healthy subject and patients. However, in patients, pain might be of greater influence due to joint contractures. The obtained results, however, show large differences between healthy boys and DMD patients, which cannot be attributed solely to underperformance.

Despite these limitations, we think this study gives valuable and objective insights in UE function and activity level of boys and men with DMD, which are of great clinical importance for the selection and evaluation of suitable interventions.

## Conclusions

The decline of muscle functions precedes the decline in performance of UE activities, and therefore may play a role in early detection of UE limitations. Early detection can have important clinical implications as it allows for starting interventions, such as contracture prevention and physical exercise training, timely and minimize functional decline. Increased sEMG levels demonstrate that DMD patients use more of their muscle capacity compared to healthy subjects to perform daily activities. This might result in increased fatigability, which should receive attention in clinical practice as this is an important determinant of quality of life. Active maximal joint angles are highly related to functional scales, therefore preserving the full range of motion is important in daily life. Monitoring active joint angles can help to select appropriate interventions timely, to minimize UE decline. Finally, the results of this study can be used for the development of new composite outcome measures for clinical trials, that not only aim at the ICF activity level, but also on the ICF level of body functions and structures.

## Abbreviations

DMD: Duchenne Muscular Dystrophy
ICF: International Classification of Functioning, Disability and Health
MVIC: Maximal voluntary isometric contraction
PUL: Performance of Upper Limb
sEMG: Surface electromyography
UE: Upper extremity
